# Completion Small Bowel Capsule Endoscopy after Device-assisted Enteroscopy in Management of Patients with Small Bowel Disease

**DOI:** 10.1055/a-2868-4670

**Published:** 2026-06-08

**Authors:** Sandeep Sihag, Edric Leung, Conor Costigan, Deirdre McNamara

**Affiliations:** 1 Gastroenterology Department 57976 Tallaght University HospitalDublinLeinsterIreland; 2 Trinity Academic Gastroenterology Group 8809 Trinity College DublinDublinLeinsterIreland

**Keywords:** endoscopy small bowel, capsule endoscopy, small bowel endoscopy, small intestinal bleeding

## Abstract

**Background and study aims** Diagnostic yields of small bowel capsule endoscopy (SBCE) and device-assisted enteroscopy (DAE) are known to be similar. SBCE is normally recommended as the initial diagnostic test, with DAE reserved for therapeutic indications. As most DAE procedures are not pan-enteric, a completion capsule may be of benefit to identify residual or overlooked disease. Our aim was to assess the clinical utility of completion SBCE performed following DAE.

**Patients and methods** A retrospective analysis of completion capsules post double balloon enteroscopy (DBE) from a single referral center was performed. Cases were identified by cross-referencing endoscopy and capsule databases. A completion capsule was defined as a capsule endoscopy requested on the enteroscopy report and performed after DAE without a new clinical indication. Main outcome measures included diagnostic yield of completion SBCE and impact on management.

**Results** Of 350 DBE patients, 70 (21%) underwent a completion SBCE, 27 of the 70 (39%) on the same day as DBE. In all, 39 of the 70 (56%) had undergone SBCE before DBE. Both completion rate (91%; 64/70) and diagnostic yield (43%; 30/70) surpassed the European Society of Gastrointestinal Endoscopy standards. A positive completion capsule resulted in a change in management in 80% of patients (24/30).

**Conclusions** Completion SBCE is feasible and effective, detecting and localizing clinically significant disease in over one-third of cases, while also reducing the need for further investigations in those with negative studies.

## Introduction

Introduction of small bowel capsule endoscopy (SBCE) and device-assisted enteroscopy (DAE) led to a paradigm shift in the diagnosis and management of small-bowel disease. Although SBCE offers a noninvasive way to visualize the small-bowel mucosa, DAE offers direct mucosal small-bowel assessment and acquisition of targeted biopsies and enables delivery of therapeutic interventions. Since being first described by Yamamoto et al. over two decades ago, 1 DAE techniques have evolved. However, despite these advances, DAE often fails to provide complete small-bowel visualization in a single procedure. In contrast, SBCE allows for complete small-bowel evaluation but lacks the ability for intervention.

In clinical practice, as a noninvasive diagnostic test, SBCE is usually performed first, followed by DAE when targeted therapy is required for diagnosis or treatment. Recent European Society of Gastrointestinal Endoscopy (ESGE) guidelines support use of SBCE prior to DAE, because there are improved lesion detection rates and reduced miss rates when enteroscopy is performed after SBCE. They also suggest DAE should be performed in over 75% of patients with an appropriate indication on capsule; those with both P1 and P2 lesions according to the Saurin classification for gastrointestinal bleeding, those with a suspicion of Crohnʼs disease on SBCE for biopsy, those with a suspicion of small-bowel tumor for biopsy and/or tattooing, when a submucosal mass is detected, and for inherited polyposis syndromes when polypectomy is indicated. 2

Although SBCE followed by DAE is the preferred pathway for the majority of small-bowel presentations, there are scenarios in which DAE may be performed first, a direct-to-DAE approach. Accepted indications for a direct-to-DAE approach include suspected small-bowel bleeding (occult or overt) when SBCE is not available or is contraindicated or for endoscopic therapy of known small-bowel bleeding and in selected cases of ongoing overt obscure gastrointestinal bleeding. In addition, clarification/reassessment of jejunal or proximal ileal Crohn’s disease, suspected on radiological tests or prior SBCE, is an indication for a direct DAE, as is the need for endoscopic therapy of established Crohn’s disease. A direct-to-DAE approach can also be considered when tissue sampling or marking of a possible small-bowel malignancy is required after identification on cross-sectional imaging or for polypectomy in patients with inherited polyposis syndromes with significant lesions again identified on imaging. Similarly, in patients with known celiac disease, enteroscopy and biopsy to diagnose or monitor refractory cases can also be performed without need for SBCE first. Finally, DAE foreign body retrieval, again localized on imaging, should not necessitate a prior capsule endoscopy. 3 4

The rationale for performing a completion capsule post DAE is two-fold: disease mapping and revalidation. Disease mapping completion capsule would be an advantage in cases where the DAE is positive, but there is a clinical suspicion of diffuse or multifocal disease. It could identify additional lesions/pathology distal to the limit of DAE insertion, thereby supporting early clinical decision-making including additional interventions or changes in therapeutic options. Second, a revalidation completion capsule performed when the DAE is negative and there is a strong clinical suspicion of small-bowel disease could identify either overlooked pathology or disease distal to limit of insertion, again facilitating early clinical decision-making, or providing reassurance when the capsule is negative, ruling out significant small-bowel disease ( Table 1 ).

**Table 1:** Rationale for completion capsule.

Completion capsule type	Index DAE result	Indications
Disease mapping	Positive	Angiodysplasia Other vascular lesions Polyposis syndrome Crohn’s disease Refractory enteropathy
Revalidation	Negative	Positive small-bowel radiology Overt suspected small-bowel bleeding Positive index capsule

DAE, device-assisted enteroscopy.

There are few data on the potential role of completion capsule endoscopy after DAE. Our study explored the value of completion capsule endoscopy post DAE in a variety of clinical scenarios.

## Patients and Methods

This study was performed as a service evaluation at Tallaght University Hospital, Dublin, Ireland. The Medical Ethics & Research Committee waived the requirement for full-study ethical approval due to the retrospective design of the study and negligible risks to patients.

Consecutive double balloon enteroscopies (DBEs) were identified and evaluated retrospectively from our endoscopy database over a 30-month period. The DBE list was then cross-checked against our SBCE database. Patient demographics, clinical details, indication for DBE, prior SBCE or cross-sectional imaging, DBE procedure data, DBE findings, and intervention as well as completion SBCE data were recorded. A completion capsule was defined as a capsule endoscopy requested on the enteroscopy report and performed after the DAE, without a new clinical indication. Patients were excluded if the SBCE was performed after the enteroscopy for new or worsening symptoms, or when a completion SBCE was requested but performed in an external institution or if bidirectional or prior pan intestinal DAEs had been performed. Positive completion SBCE was defined as any clinically significant finding that was either overlooked during DBE or was distal to maximal point of insertion. Clinically significant disease or findings included P2 vascular lesion, active bleeding, significant enteritis (Lewis score > 135), polyps, or suspicious masses.

Primary outcomes were diagnostic yield and impact on management of completion SBCEs. Secondary outcomes included technical efficacy, including complete transit, adequate visualization, and identification of tattoos applied during DAE to mark the distal insertion point. Where appropriate, outcomes were compared among groups, using a Fisher exact test, and odds ratios (ORs) were calculated as appropriate; *P* < 0.05 was considered significant.

## Results

Of the 350 DBE patients, 70 (21%) underwent completion SBCE. Mean age was 59 years (range 17–88), with 45 (60%) male patients. The indication for DBE was angiodysplasia ( *N* = 27; 38%), overt suspected small-bowel bleeding ( *N* = 13; 18%), abnormal small-bowel radiology ( *N* = 20; 29%), significant enteritis on capsule requiring biopsy ( *N* = 6; 9%), known Crohn’s disease ( *N* = 2; 3%), and small-bowel polyps in polyposis syndrome patients ( *N* = 2; 3%). Most DBEs were anterograde ( *N* = 67; 96%), and complete small-bowel assessment was not performed on any enteroscopy. Mean depth of insertion was 300 cm (range 40–600 cm) and 123 cm (range 10–200 cm) for ADBE and RDBE, respectively. A tattoo was placed at the maximal insertion point on 69 of 70 DBEs (99%). Overall, 27 of 70 DBEs (39%) were positive and 24 of 27 (93%) included an intervention including biopsy or marking of a lesion.

In all, 39 of 70 (56%) had SBCE before proceeding to DAE. The indication for DAE in those who had a prior SBCE was small-bowel bleeding (majority angiodysplasia), significant enteritis (Lewis score >250), small-bowel polyps, ongoing overt suspected small-bowel bleeding despite negative capsule, and persistent abnormal radiology despite negative capsule in 27 of 39 (69%), 6 of 39 (15%), 2 of 39 (5%), 2 of 39 (5%), and 2 of 39 (5%), respectively. Of the 31 of 70 (44%) who did not have SBCE before proceeding to DBE, 18 of 31 (58%) had abnormal imaging, 11 of 31 (36%) had severe suspected overt small-bowel bleeding, and 2 (6%) had known Crohn’s disease. As expected, those with SBCE prior to DAE were almost four times more likely to have a positive finding on DAE (20 of 39 [51%] vs. 7 of 31 [23%]; OR 3.6, 95% confidence interval [CI] 1.26–10.3, *P* = 0.02).

**Fig. 1:**
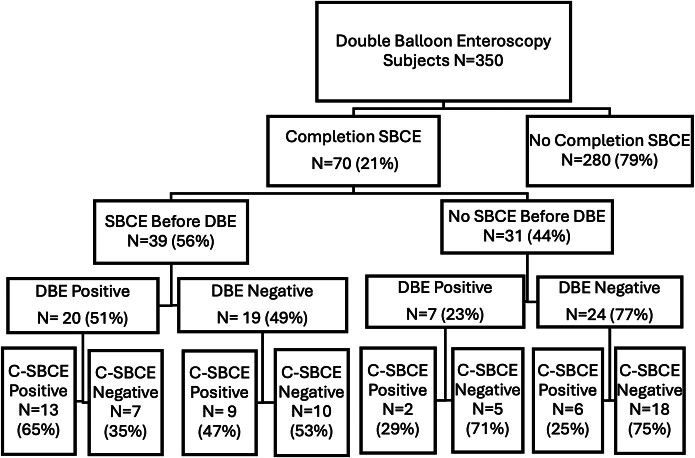
Study population.

In 27 of 70 patients (39%), completion SBCE was done the same day as DBE. The remaining 43 of 70 (61%) were performed on average 12 weeks later (range 1-36 weeks). Capsule studies were complete and reached the colon in 64 of 70 patients (91%). The completion rate was similar for capsules performed on the day of enteroscopy and after an interval 24 of 27 (89%) vs. 40 of 43 (93%). Completion SBCEs identified significant findings in 30 of 70 (43%): 14 of 30 (47%) angiodysplasias, 7 of 30 (23%) active bleeding without a visible source, 5 of 30 (17%) significant enteritis, and 4 of 30 (13%) small-bowel polyps ( Fig. 1 ). As expected in a cohort who had already undergone either SBCE or cross-sectional imaging, there were no unexpected or alternative clinically significant diagnoses or pathologies identified on completion capsules. Overall, 24 of 30 patients required changes in management, including starting long-acting octreotide formulations in 6 patients, change of Crohn’s medications in 2, 14 patients underwent a further DBE and intervention, and 2 patients were referred for surgery.

Of interest, patients with an indication of abnormal small-bowel radiology for DBE were eight times less likely to have a positive completion capsule (2/20 [10%] vs. 28/50 [56%], OR 8, 95% CI 1.7–37, *P* = 0.01). Furthermore, patients with a positive DBE were twice as likely to have a positive completion capsule (15/27 [56%] vs. 15/43 [35%], OR 2.3, 95% CI 1–6, *P* = 0.1) ( Table 2 ). In addition, patients who had a capsule endoscopy prior to their DBE were also more likely to have a positive completion capsule (22/39 [54%] SBCE prior to DBE vs. 8/31 [26%] without a SBCE before the DBE, *P* < 0.01).

**Table 2:** Diagnostic yield of completion capsule endoscopy based on initial DBE indication.

	Indication for DBE	Completion SBCE findings (DY %)
Angiodysplasia	27	14 (52%)
Suspected overt bleed	13	7 (54%)
Abnormal radiology	20	2 (10%)
Enteritis	6	4 (67%)
Polyps	1	1 (100%)
Crohn’s disease	2	1 (50%)
Polyposis syndrome	1	1 (100%)

DBE, double balloon enteroscopy; SBCE, small bowel capsule endoscopy.

Of patients with a limit of insertion tattoo placed at DBE, 46 of 69 (67%) were visible and identified on completion capsule; in 20 of 30 (67%), positive completion capsules and 26 of 40 (65%), negative completion capsules. Of the positive completion capsules, lesion localization was proximal to the tattoo in 13 of 20 (65%); 8 of 13 (62%) angiodysplasias, 2 of 13 (15%) active bleeding without a visible source, 2 of 13 (15%) small-bowel polyp, and 1 of 13 (8%) significant enteritis.

Overall, a positive completion capsule ( *N* = 30) resulted in a change in management in 24 of 30 patients (80%). Long-acting octreotide formulations were initiated in 6, Crohn’s medications were changed in 2, 14 subjects underwent a further DBE and intervention, and 2 were referred for surgery.

## Discussion

To our knowledge, this is the first study to describe utility of completion SBCE in a cohort of enteroscopy patients. Overall technical outcomes for completion capsules were good reaching updated ESGE quality performance measures, with a completion rate of 91% and a high diagnostic yield of 43%. These outcomes were similar for those performed immediately after enteroscopy and performed at a later date. In general, being able to perform small-bowel capsule post DBE on the same admission offers obvious advantages, including reduced patient travel and inconvenience, reduced cost and emissions, and improved timeliness.

As expected, the cohort of patients who had SBCE prior to enteroscopy had a higher yield on completion capsule (54%), as did those with a positive DBE (56%). The majority had diffuse vascular lesions or enteritis that was either overlooked during the DBE or identified distal to the limit of insertion. Despite this, the yield in direct-to-DBE cases (no SBCE before DBE) and in those with a negative DBE was still substantial at 26% and 35%, respectively.

In the group without a capsule before proceeding to enteroscopy, the majority (20/31) had abnormal radiology. In this cohort (no prior SBCE), a completion capsule endoscopy was performed in those with a negative DBE to ensure there was no significant disease distal to the limit of insertion (tattoo) warranting either an RDBE or other intervention, because in all of these patients, initial radiology was suggestive of disease, whereas for those who had positive DBE ( *n* = 3), completion capsules were undertaken to map the condition including extent of enteritis or presence of additional polyps. Of interest, these patients were eight times less likely to have a positive completion capsule (2/20 [10%] vs. 28/50 [56%], OR 8, 95% CI 1.7 to 37, *P* = 0.01).

Most importantly a positive completion capsule resulted in a management change in 80% of cases, confirming clinical utility of the approach, including further DBE or medical intervention to treat residual disease. Similarly, a negative completion capsule was also clinically relevant because it supported a conservative approach, suggesting that intervention with DBE was sufficient.

In particular, 90% of patients (18/20) referred with significant abnormalities on small-bowel radiology had a negative completion capsule and required no further intervention or investigation.

Of interest in our cohort, despite reviewing all capsule videos, the tattoos placed at enteroscopy to mark the limit of insertion were identifiable only in 67% of capsules. Similarly, of the positive completion capsules where the DBE tattoo was seen ( *N* = 20), significant abnormalities were detected in 13 (65%) proximal to the tattoo, the majority were vascular lesions. Both of these findings highlight the possibility of overlooked lesions/pathology for both capsule and enteroscopy procedures. The true false-negative rate for either procedure is not well established and clinicians should be aware of the possibility.

Although our study shows that completion capsule post enteroscopy is feasible and effective, there are obvious limitations. First, as a tertiary national referral center for enteroscopy and capsule endoscopy, access to capsule endoscopy is not a limitation, which may not be the case in all centers. The ready availability of capsule may also bias our approach because we can facilitate completion capsules. Overall, a completion capsule was performed in 21% of our DBE cohort. However, if anything, this would suggest overuse and negative effect on actual yield and clinical relevance, where the true value of completion capsule is underestimated in our study. Also, our cohort would include patients from locations without timely access to capsule endoscopy, which could overestimate the number referred directly for DBE and, therefore, the need for completion capsules in general. Having said that, 56% of our cohort had SBCE prior to their DBE and of those without a capsule prior to the DBE, 58% had significant positive findings on dedicated small-bowel imaging, a minority only were referred as a result of capsule availability or access issues.

The retrospective nature of our study, as expected, also limits its interpretation, because we have no information on the cohort who did not have a completion capsule. Despite this, we believe our study suggests clinical value and utility for completion capsule in small-bowel diagnostics and patient management that warrants further more detailed investigation.

## Conclusions

In conclusion, completion SBCE after DAE is feasible and effective, with a high diagnostic yield and clinical utility.

## References

[1] Yamamoto H Sekine Y Sato Y Total enteroscopy with a nonsurgical steerable double-balloon method Gastrointest Endosc 2001 53 216 220 11174299 10.1067/mge.2001.112181

[2] Sidhu R Shiha M G Carretero C Performance measures for small-bowel endoscopy: a European Society of Gastrointestinal Endoscopy (ESGE) quality improvement initiative – update 2025 Endoscopy 2025 57 366 389 39909070 10.1055/a-2522-1995

[3] Pennazio M Rondonotti E Despott E J Small-bowel capsule endoscopy and device-assisted enteroscopy for diagnosis and treatment of small-bowel disorders: European Society of Gastrointestinal Endoscopy (ESGE) guideline – update 2022 Endoscopy 2023 55 58 95 36423618 10.1055/a-1973-3796

[4] Rondonotti E Spada C Adler S Small-bowel capsule endoscopy and device-assisted enteroscopy for diagnosis and treatment of small-bowel disorders: European Society of Gastrointestinal Endoscopy (ESGE) technical review Endoscopy 2018 50 423 446 29539652 10.1055/a-0576-0566

